# Diversity in Phenotypes Associated With Host Persistence and Systemic Virulence in *Streptococcus sanguinis* Strains

**DOI:** 10.3389/fmicb.2022.875581

**Published:** 2022-04-18

**Authors:** Livia A. Alves, Geovanny C. Salvatierra, Victor A. Freitas, José F. Höfling, Débora C. Bastos, Thaís L. S. Araujo, Renata O. Mattos-Graner

**Affiliations:** ^1^Department of Oral Diagnosis, Piracicaba Dental School, State University of Campinas, Piracicaba, Brazil; ^2^Department of Biosciences, Piracicaba Dental School, State University of Campinas, Piracicaba, Brazil; ^3^São Leopoldo Mandic Medical School, Campinas, Brazil; ^4^Department of Biochemistry, Institute of Chemistry, University of São Paulo, São Paulo, Brazil

**Keywords:** *Streptococcus sanguinis*, biofilm, cardiovascular infections, exopolysaccharides, eDNA, complement system, endothelial cells

## Abstract

*Streptococcus sanguinis* is a pioneer commensal species of dental biofilms, abundant in different oral sites and commonly associated with opportunist cardiovascular infections. In this study, we addressed intra-species functional diversity to better understand the *S. sanguinis* commensal and pathogenic lifestyles. Multiple phenotypes were screened in nine strains isolated from dental biofilms or from the bloodstream to identify conserved and strain-specific functions involved in biofilm formation and/or persistence in oral and cardiovascular tissues. Strain phenotypes of biofilm maturation were independent of biofilm initiation phenotypes, and significantly influenced by human saliva and by aggregation mediated by sucrose-derived exopolysaccharides (EPS). The production of H_2_O_2_ was conserved in most strains, and consistent with variations in extracellular DNA (eDNA) production observed in few strains. The diversity in complement C3b deposition correlated with the rates of opsonophagocytosis by human PMN and was influenced by culture medium and sucrose-derived EPS in a strain-specific fashion. Differences in C3b deposition correlated with strain binding to recognition proteins of the classical pathway, C1q and serum amyloid protein (SAP). Importantly, differences in strain invasiveness into primary human coronary artery endothelial cells (HCAEC) were significantly associated with C3b binding, and in a lesser extent, with binding to host glycoproteins (such as fibrinogen, plasminogen, fibronectin, and collagen). Thus, by identifying conserved and strain-specific phenotypes involved in host persistence and systemic virulence, this study indicates potential new functions involved in systemic virulence and highlights the need of including a wider panel of strains in molecular studies to understand *S. sanguinis* biology.

## Introduction

*Streptococcus sanguinis* is a commensal member of the oral microbiome of humans, abundant in several oral sites, such as dental biofilms, its major oral niche (Huttenhower et al., 2012; Bin et al., 2018). *S. sanguinis* is acquired early in life, emerging with the eruption of primary teeth (Carlsson et al., 1970; Caufield et al., 2000; Jiménez et al., 2005). There is evidence that *S. sanguinis* contributes to microbiome homeostasis, as it shows increased abundance in children and in adults with good oral health, when compared to caries-affected individuals (Ge et al., 2008; Giacaman et al., 2015). In addition, this species can inhibit the growth of pathogenic species involved in dental caries (*Streptococcus mutans*) and periodontal diseases (*Porphyromonas gingivalis*), through the production of hydrogen peroxide (Kreth et al., 2017). The ability of this species to initiate tooth colonization and to contribute to biofilm maturation remains to be better understood but involves the production of extracellular DNA (eDNA) and sucrose-derived exopolysaccharides (Kreth et al., 2017; Bin et al., 2018). Genomic analysis of *S. sanguinis* strain SK36 indicates a large number of surface proteins which could potentially interact with salivary components, as well as with plasma and tissue components present in oral and extra-oral surfaces (Xu et al., 2007; Bin et al., 2018; Nobbs and Kreth, 2019). There is also evidence that *S. sanguinis* is tolerated by innate and adaptive immune functions when compared to other oral streptococci (Salam et al., 2006; Alves et al., 2019). Despite the beneficial role of this species in the oral cavity, *S. sanguinis* is commonly associated with opportunist cardiovascular infections in susceptible hosts (von Reyn et al., 1981; Douglas et al., 1993; di Filippo et al., 2006; Nakano et al., 2006), which seems to be associated with its fitness and persistence in human blood, resistance to PMN killing and invasion of endothelial cells (Paik et al., 2005; Yamaguchi et al., 2006; Morita et al., 2014; Ge et al., 2016; Sumioka et al., 2017; Martini et al., 2020; Zhu et al., 2021). There is, however, limited information on functional diversity among *S. sanguinis* strains. Comparative analyses of a range of 20–25 *S. sanguinis* genomes available in public databases show that genes involved in biofilm and/or systemic virulence are present in most strains, although variation in gene-associated phenotypes remains to be addressed (Kreth et al., 2017; Baker et al., 2018). In the present study, we explored the diversity of multiple phenotypes associated with biofilm formation, host persistence and/or systemic virulence in a collection of nine *S. sanguinis* strains isolated from the oral cavity or from the bloodstream with partial genomes available in the GenBank. Planktonic growth and biofilm formation at different culture conditions and/or exposure to human saliva and sucrose-mediated aggregation and kinetics of the production of H_2_O_2_ and eDNA were assessed. In addition, a screening of the bacterial interactions with plasma and/or extracellular matrix (ECM) components was compared to phenotypes of susceptibility to complement deposition and opsonophagocytosis by human peripheral neutrophils (PMN) and with invasion into primary human coronary artery endothelial cells. Our results revealed high variation in phenotypes associated with biofilm formation and/or systemic virulence, further indicating novel potential mechanisms contributing to host persistence and systemic virulence.

## Materials and Methods

### Bacterial Strains and Culture Conditions

Strains used in this study are shown in Table 1 (Kilian and Holmgren, 1981; Kilian et al., 1989; Bishop et al., 2009). The *S. mutans* strains UA159 and OMZ175 were used as reference in specific assays (Ajdić et al., 2002; Abranches et al., 2011). These strains were routinely grown from frozen stocks in brain heart infusion (BHI) agar (BD Difco, MI, United States) at 37°C in a 10% CO_2_ atmosphere. For phenotypic analyses, overnight (18 h) cultures obtained in BHI with adjusted absorbances were diluted into fresh BHI supplemented or not with 10% of human saliva (BHIS) or in chemically defined medium (CDM; Biswas et al., 2007), and incubated at 37°C in 10% CO_2_ (90% air) or in aerobiosis under rotational aeration (160 rpm).

**TABLE 1:** Strains used in this study.

Strain	Isolation site	Genome accession n*^o^*.*	Source and/or references
***Streptococcus sanguinis***			
SK36	Dental biofilm	GCA_000960035.1	ATCC 10556; Xu et al., 2007
SK49^†^	Dental biofilm	GCA_000212815.1	Mogens Kilian; Kilian et al., 1989
SK72^†^	Dental biofilm	GCA_000192185.1	Mogens Kilian; Kilian et al., 1989
SK115^†^	Dental biofilm	GCA_000192205.1	Mogens Kilian; Kilian et al., 1989
SK160^†^	Dental biofilm	GCA_000192245.1	Mogens Kilian; Kilian et al., 1989
SK330^†^	Oral cavity	GCA_000195025.1	Mogens Kilian; Bishop et al., 2009
SK353^†^	Oral cavity	GCA_000191085.1	Mogens Kilian; Bishop et al., 2009
SK678^†^	Blood	GCA_000212835.1	Mogens Kilian; Bishop et al., 2009
SK1056^†^	Blood	GCA_000191125.1	Mogens Kilian; Bishop et al., 2009
***Streptococcus mutans***			
UA159	Oral cavity, child with active caries	GCA_000007465.2	ATCC; Ajdić et al., 2002
OMZ175^++^	Dental biofilm		Jacqueline Abranches; Abranches et al., 2011

^†^Provided by Dr. Mogens Kilian, Aarhus University, Denmark. ^++^Provided by Dr. Jacqueline Abranches, University of Florida, United States. *Genomes available at NCBI-GenBank (https://www.ncbi.nlm.nih.gov/genome/genomes/).

### Collection of Saliva, Serum, and Blood Samples

Saliva samples were collected from a healthy reference volunteer, according to a protocol previously approved by the Ethics Committee of the Piracicaba Dental School, State University of Campinas (CEP/FOP-UNICAMP; protocol No. 3.365.892). The reference volunteer (30 years of age) was a non-smoker, with good general, and oral health; the absence of active lesions of dental caries or periodontal disease was verified in clinical intra-oral examination performed by a dental professional (VAF). Whole saliva samples were collected at morning, after at least 2 h of fasting. Briefly, salivation was stimulated by parafilm chewing, and then whole saliva was collected in glass tubes maintained on ice bath. Saliva samples were then clarified by centrifugation (40,000 × *g*; 15 min, 4°C), sterilized by filtration under vacuum through filter with pore sizes of 0.22 μm diameter (Millipore Express™ Plus, Merck KGaA, Germany), and stored at −70°C until use. Serum and blood samples were also collected from the reference healthy volunteer (Alves et al., 2020) by using a standard protocol, as previously approved by the Ethics Committee of the Piracicaba Dental School, State University of Campinas (UNICAMP) (CAAE: 83140418.0.0000.5418).

### Bacterial Growth Curves

Growth curves of *S. sanguinis* strains were determined in BHI. Briefly, 18 h cultures with adjusted A_550 nm_ (0.08) were diluted (1:6) into fresh BHI (Schott Duran, Germany) and incubated at 37°C under the aerobiosis (in a shaker incubator, China) or in 10% CO_2_ atmosphere. The absorbances of cultures were then determined at each 1 h over an 8-h period. Three assays were performed for each strain at each atmospheric condition.

### Biofilm Formation Assays

Biofilms were formed in 96-well flat bottom polystyrene plates (CralPlast) and quantified as previously described (Camargo et al., 2018), with minor modifications. Briefly, 18 h BHI cultures with adjusted absorbances (A*_5_*_50 nm_ of 0.3) were diluted (1:10) into 5 ml of fresh medium (BHI or BHIS) supplemented with 1% sucrose. Diluted cultures were transferred to 96-well plates (200 μl/well) and incubated (37°C) under aerobiosis (Thermo Shaker Incubator, China) for 18 h. Next, the culture fluids were removed from each well and the plates were gently washed three times with the distilled water to remove weakly adhered cells. The biofilms were then stained with 1% crystal violet for 30 min at room temperature. Afterward, the stain was eluted in ethanol from biofilms, and A_575 nm_ of the eluates was determined and expressed as indirect measures of biofilm biomass. Planktonic growth (A_550 nm_) was also assessed in the same bath cultures to control the bacterial growth.

### Biofilm Formation on Glass Slides and Scanning Electron Microscopy

The initial phases of biofilm formation by *S. sanguinis* strains were investigated by scanning electron microscopy (SEM) as previously described (Moraes et al., 2014), with minor modifications. Briefly, glass slides (10 mm × 10 mm) were treated with 1 ml of sterile saliva (18 h at 4°C) in 24-well microplates (Corning, NY, United States). Slides were then incubated with strain cultures in BHI supplemented with 1% sucrose, during 4 h at 37°C under rotational aeration (at 160 rpm). Afterward, slides were washed three times with 0.9% saline, fixed with 2.5% glutaraldehyde (Sigma-Aldrich, United States) and dehydrated in ethanol. Specimens were then sputter coated with gold and analyzed in a scanning electron microscope (JSM5600 LV; JEOL, Japan). Representative images of the specimens were obtained under magnification of 5,000× and 10,000×. Slides incubated with uninoculated medium were used as negative controls.

### Quantification of Extracellular DNA

Amounts of eDNA were measured by the quantitative PCR (qPCR) as described elsewhere (Camargo et al., 2018). Briefly, culture supernatants were obtained from 1 ml of cultures in BHI medium (aerobiosis at 37°C) at the late log phase of growth (A_550_ of 0.7) by centrifugation (twice at 16,000 × *g* at 4°C for 10 min), followed by filtration through polyethersulfone membranes (0.22-μm pore diameter; Millipore, United States) for the removal of remaining cells. The qPCR was performed in a StepOne real-time PCR system (Life Technologies, United States) with volumes of 1 μl of culture supernatants mixed with 3.4 μl molecular-grade water, 5 μl of Power SYBR green PCR master mix (Thermo Fischer Scientific, United States), and 0.3 μl of a 10 mM stock solution of each primer for gene encoding 16S rRNA (Forward: 5′-CGTAAACGATGAGTGCTAGGTG-3′; Reverse: 5′-TAGAGCGGTCAGAGGGATGT-3′). The qPCR cycling conditions were 95°C for 10 min followed by 40 cycles of 95°C for 15 s, 58°C for 15 s, and 72°C for 30 s. The DNA concentration was calculated based on average threshold cycle values against a 10-fold dilution series of purified SK36 genomic DNA in the same medium. Sterile culture medium was used as a negative control in qPCRs. Three independent experiments were performed in duplicate.

### Bacterial Binding to Complement Proteins

Bacterial binding to complement proteins [C3b, C1q, serum amyloid protein (SAP), C4b-binding protein (C4BP), and factor H (FH)] were determined as previously described (Alves et al., 2016, 2020) with some modifications. Briefly, approximately 10^7^ CFU from mid-log phase cultures (A_550 nm_ 0.3) were harvested by centrifugation (10,000 × *g*, 4°C), washed twice with PBS (pH 7.4) and resuspended in 20 μl of 20% serum in PBS. After incubation (37°C, 30 min), cells were washed twice with PBS-Tween 0.05% (PBST) and incubated with specific antibodies diluted in PBST, which included fluorescein isothiocyanate (FITC)-conjugated polyclonal goat anti-human C3 IgG antibody (1:300; on ice for 40 min) (ICN, United States), FITC-conjugated polyclonal goat anti-human C1q (1:300; 37°C for 60 min) (LSBio, United States) (Domenech et al., 2013), FITC-conjugated polyclonal anti-human SAP (1:200; 37°C for 60 min) (LSBio, United States) (Yuste et al., 2007), or FITC-conjugated polyclonal rabbit anti-human C4BP (1:225 in PBST; 25°C for 60 min) (LSBio, United States) (Domenech et al., 2013). Factor H was detected by incubation with goat anti-human FH IgG (1:100, 37°C for 30 min) (Calbiochem) followed by incubation with FITC-conjugated anti-goat IgG (1:1,000; 4°C, for 40 min). After antibody probing, cells were washed twice with PBST and fixed in 3% paraformaldehyde in PBS for flow cytometry analyses using a FACSCalibur flow cytometer (BD Biosciences, United States). A total of 25,000 bacterial cells were gated using forward and side scatter parameters. Results were expressed as the geometric mean fluorescence intensity (MFI) of positive cells for each complement protein and as fluorescent index (FI) determined by multiplying MFI by the percentage of positive cells (Alves et al., 2016). Control samples included cells treated only with PBS instead of serum. In addition, heat-inactivated sera (56°C for 20 min) were applied as negative controls in preliminary experiments with each of the tested strains and antibodies, showing minimal effects on comparative analyses of the strains.

### PMN Isolation and Phagocytosis Assays

Human PMN were isolated from the fresh heparinized blood samples as previously described, with modifications (Alves et al., 2016), using centrifugation over a double gradient composed of 1,119 and 1,083 density Histopaque (Sigma-Aldrich, United States); red blood cells were removed by hypotonic lysis. Isolated PMN was suspended in RPMI 1640 [GIBCO (Thermo Fisher Scientific, United States), Life Technologies, NY, United States] medium supplemented with inactivated 10% fetal bovine serum. Cell viability (>98%) and purity (>95%) were monitored by the trypan blue exclusion and May-Grunwald Giemsa staining, respectively. Bacteria used in the phagocytosis assays were previously labeled with FITC as described elsewhere (Alves et al., 2016). Briefly, 500 μl of bacterial strains (A_550 nm_ 0.3) were washed twice in PBS, suspended in FITC (Sigma) solution [1.7 mg/ml in carbonate buffer (Na_2_CO_3_ 0.15 M, 0.9% NaCl; pH 9)], and the suspensions were incubated for 1 h (shaking at room temperature in the dark). Next, cells were harvested and washed three times with PBST, and aliquots were stored overnight in 10% glycerol at −70°C.

To analyze serum-mediated phagocytosis, aliquots containing 10^7^CFU of FITC-labeled bacteria were added to wells of 96-well plates containing 2 × 10^5^ PMNs in 50 μl of RPMI medium supplemented with 20% of human serum to a multiplicity of infection (MOI) of 200 bacteria per PMN. Plates were then incubated for 5 min (37°C, 10% CO_2_, gentle shaking), and cells were fixed by addition of 100 μl/well of 3% paraformaldehyde. To assess serum-independent phagocytosis, parallel assays were performed in the absence of human serum. PMNs were then analyzed using FACSCalibur (BD Biosciences, United States), and the frequency of phagocytosis expressed as the number of PMN cells with intracellular bacteria, within a total of 10,000 PMN analyzed (Alves et al., 2016).

### Bacterial Binding to Plasma and Extracellular Matrix Human Proteins

The binding of bacteria to plasma and/or ECM proteins was analyzed as described elsewhere (Agarwal et al., 2013) with modifications. Briefly, black polystyrene 96-well microtiter plates were treated (18 h at 4°C) with each human protein [plasma fibronectin, plasma fibrinogen, plasma plasminogen, or type I collagen from human fibroblast (Sigma-Aldrich, United States); 5 μg/ml in PBS pH 7.2]. Afterward, the plates were washed (three times with PBST) and incubated (2 h at room temperature) with blocking solution (50 mM Tris–HCl – pH 8, 150 mM NaCl, 0.1% Tween 20, 3% fish gelatin). Next, 100 μl of suspensions of FITC-labeled bacteria in carbonate buffer (0.15 M NaCl, 0.1 M Na_2_CO_3_; pH 9.6) (containing 1 × 10^8^ ufc) was added per well, and plates incubated during 1 h at 37°C. Plates were then washed three times with washing buffer (50 mM Tris–HCl pH 8.0, 150 mM NaCl, 0.1% Tween 20) for removal of unbound bacteria, and fluorescence signal quantified with the help of a microplate fluorescence reader (Synergy H1, BioTek, CA, United States). The control samples for each strain included wells treated with each human protein, but not incubated with FITC-labeled bacteria, and wells treated with BSA followed by the incubation with the bacterial suspensions.

### Bacterial Invasion of Primary Human Coronary Artery Endothelial Cells

Primary HCAEC cells were obtained from Lonza and cultivated in basal medium (EBM-2, Lonza, United States) supplemented with EGM-2MV (Lonza, United States). Invasion assays were performed as described elsewhere (Abranches et al., 2011), with modifications. Briefly, HCAEC cells were seeded (1 × 10^5^ cells/well) in 24-well culture plates (Corning, NY, United States), medium removed, and cells were washed three times with pre-warmed HEPES buffer. Next, HCAEC were added of 1 ml of bacterial suspensions containing 1 × 10^7^ cfu in antibiotic-free EGM-2 medium supplemented with 2% of fetal bovine serum (to a MOI of 100:1), and incubated (5% CO_2_, 37°C) during periods of 2, 4, and 6 h. After the incubation periods, culture supernatants were removed and the HCAEC cells were washed three times with pre-warmed HEPES buffer and incubated with EBM-2 medium supplemented with penicillin G (50 μg/ml) and gentamycin (300 μg/ml) during 1 h for killing of extracellular bacteria. Afterward, culture fluids were removed for plating onto BHI (to monitor the presence of extracellular bacteria) and the HCAEC was washed with pre-warmed HEPES buffer as described, and lysed in cold ultrapure type I H_2_O at room temperature for 20 min. Serial dilutions of cell lysates were plated in BHI agar for bacterial counting after 48 h of incubation (10% CO_2_, 37°C). The counts of cfu in the initial inoculum were used as reference for calculating the percentages of invasion. To analyze the effects of human serum in bacterial invasion, similar assays were performed by incubating the HCAEC cells with bacteria previously treated (37°C, 20 min) with 20% human serum or with PBS (negative control) during 2 h.

### Data Analysis

Strain comparisons with the reference strain SK36 were performed using a non-parametric Mann–Whitney *U*-test. The flow cytometry data (MFI, FI, and frequencies of phagocytosis by PMN) were compared using Kruskal–Wallis with *post hoc* Dunn’s test or Mann–Whitney *U*-test. The Pearson correlation was used to identify associations between binding to complement proteins and/or host plasma/ECM proteins and HCAEC invasion. Differences were considered significant when a *p* value of <0.05 was obtained.

## Results

### Analysis of Growth Characteristics of *S. sanguinis* Strains at Different Atmospheric Conditions

Growth characteristics of strains were assessed by comparing growth curves in BHI medium at different oxygen tensions, including static incubation at 10% CO_2_ (90% air) (Supplementary Figures 1A,B) and culture aeration (Supplementary Figures 1C,D). Strain SK72 showed the lowest growth in both atmospheric conditions among all strains analyzed (Supplementary Figures 1B,D), whereas SK678 showed increased growth under medium aeration when compared to SK36 and other strains (Supplementary Figures 1C,D).

### Phenotypes of Biofilm Initiation on Saliva-Coated Surfaces Are Strain-Specific and Independent of Biofilm Maturation Phenotypes or Sucrose-Mediated Aggregation

**FIGURE 1:**
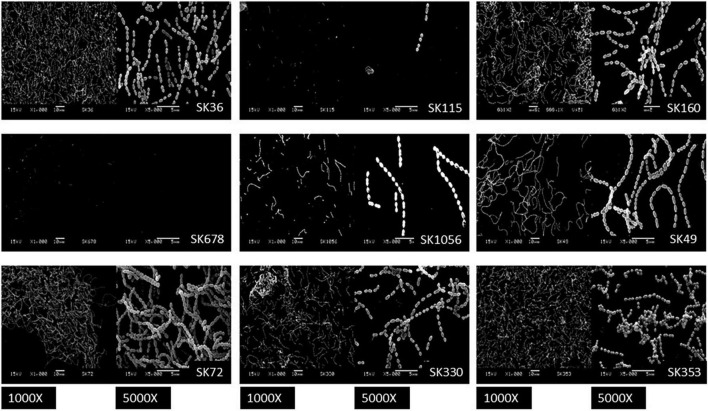
Biofilms formed by *Streptococcus sanguinis* strains on saliva-coated glass surfaces. Glass slides coated with filter-sterilized human saliva were incubated with 1:10 dilutions of cultures in brain heart infusion (BHI) supplemented with 1% sucrose (A_550 nm_ 0.3) and incubated during 4 h under aerobiosis (rotary aeration). Biofilms were then washed and processed for scanning electron microscopy analysis. Representative digital images obtained at magnification of 1,000 and 5,000 are shown for each strain, as identified in the respective panels.

**FIGURE 2:**
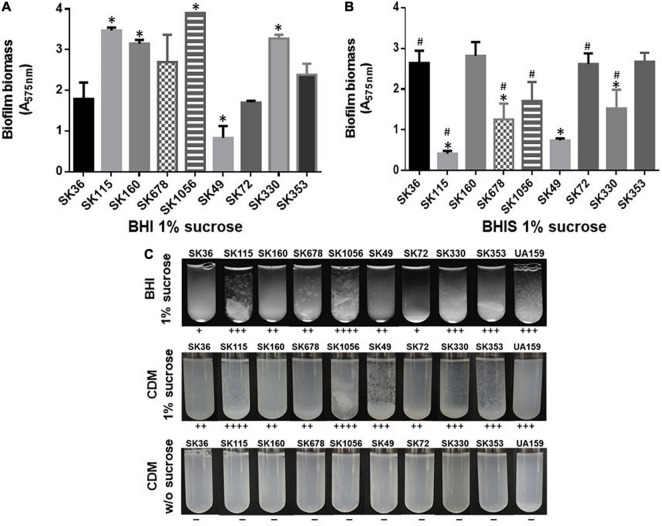
Comparisons of biofilm maturation and exopolysaccharides (EPS)-mediated aggregation in *S. sanguinis* strains. **(A)** Biomass measurements of biofilms formed during 18 h on polystyrene 96-well microtiter in BHI w/1% sucrose under aerobiosis, at 37°C. **(B)** Biomass measurements of 18 h biofilms formed under similar conditions, but in BHI w/1% sucrose supplemented w/10% human saliva (BHIS). The absorbances of ethanol eluates of biofilms stained with 1% crystal violet were expressed as relative measures of biofilm biomass (A_575 nm_). The columns represent the means of data obtained in three independent experiments performed in four replicates. Bars indicate standard deviations. Asterisks indicate significant differences in relation to the reference strain SK36 (Mann–Whitney; *p* < 0.05). Hashtags indicate significant differences in relation to biofilms formed by the same strain in BHI-sucrose medium without saliva (shown in **A**). **(C)** Bacterial aggregation in sucrose-derived EPS. Equal numbers of cells from 18 h cultures in BHI or CMD were inoculated into fresh medium supplemented or not with 1% sucrose and incubated during 18 h. Aggregation scores were determined by visual analysis of cultures and indicated below the respective images. Cells from cultures obtained in either BHI (not shown) or chemically defined medium (CDM) not supplemented with sucrose (at bottom) did not show cell aggregation.

*Streptococcus sanguinis* SK36 depends on the presence of saliva and production of eDNA to initiate biofilms (Kreth et al., 2017). Additionally, the biofilm maturation *in vitro* requires the presence of sucrose, the only substrate for glucan synthesis by the glucosyltransferase GtfP expressed by SK36 (Xu et al., 2007; Moraes et al., 2014). Here, we investigated the biofilm initiation on saliva-coated glass surfaces and biofilm maturation in the presence of sucrose using microtiter plate assays. Consistently with our previous studies (Moraes et al., 2014), SK36 was able to initiate biofilms on glass slides coated with human saliva, forming a homogeneous monolayer with spaced small micro colonies after 4 h of grow (Figure 1), and similar phenotypes were also observed in the majority of strains, except for SK115 and for the blood isolates SK678 and SK1056. We then assessed strain capacities to mature biofilms during 18 h of growth in BHI with 1% sucrose, observing biofilms with robust biomass (Figure 2A) formed by strains defective in biofilm initiation (SK115, SK678, and SK1056) (Figure 1), a trait also observed in SK160 and SK330 (Figure 2A). On the other hand, strains SK49 and SK72 showed reduced capacities to mature the biofilms in the presence of sucrose (Figure 2A). Parallel analysis of planktonic growth of cultures used for biofilm formation confirmed that reduced biofilm maturation was not due to growth defects (data not shown). We thus investigated the influence of saliva components in biofilm maturation by performing the similar assays using BHI w/1% sucrose supplemented with 10% of filter-sterilized human saliva. As shown in Figure 2B, saliva supplementation clearly reduced the biofilm maturation of SK115, SK678, SK1056, and SK330, but increased biofilm maturation by SK72. On the other hand, SK49 retained defects in biofilm formation in saliva supplemented BHI, whereas saliva supplementation did not significantly change the biofilm maturation in SK36, SK160, and SK353 (Figure 2B). Thus, the capacity of *S. sanguinis* to initiate biofilms on saliva-coated surfaces are not associated with their ability to mature biofilms in the presence of sucrose, a process that may be compromised or enhanced by salivary components in a strain-dependent fashion.

To further investigate if differences in biofilm maturation were associated with bacterial binding to sucrose-derived EPS, we analyzed profiles of bacterial aggregation in BHI and CDM media, with and without 1% sucrose. As shown in Figure 2C, strains with increased capacity to form mature biofilms (SK115, SK160, SK1056, and SK353) showed increased aggregation in BHI or CDM supplemented with sucrose when compared to SK36, SK49, and SK72. The appearance of the bacterial aggregates was, however, variable between strains. For example, strains SK115, SK678, and SK1056 formed silky flocculates in BHI-sucrose and/or CDM-sucrose, which did not resemble the clump aggregates mediated by the sucrose-derived glucan typical of cariogenic *S. mutans* strains (Alves et al., 2016; Harth-Chu et al., 2019; Figure 2C). In addition, the aggregates formed by SK160 in BHI/CDM supplemented with sucrose were numerous, but of reduced size, making then difficult to notice in the digital images of tubes shown in Figure 2C. None of the strains formed aggregates in media not supplemented with sucrose, either in CDM (Figure 2C) or in BHI (data not shown). Thus, apart from differences in aggregate structure, strains with increased capacity to mature biofilms in the presence of sucrose, also showed significant aggregation mediated by sucrose-derived EPS.

### Production of H_2_O_2_ Is a Conserved Function of *S. sanguinis* Strains and Compatible With Phenotypes of Extracellular DNA Production

**FIGURE 3:**
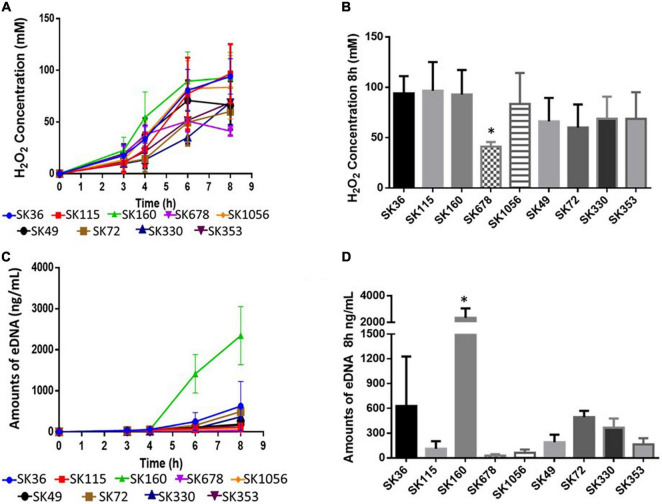
Comparisons of the production of H_2_O_2_ and eDNA by *S. sanguinis* strains. Kinetics of H_2_O_2_ and eDNA production were assessed in samples of culture supernatants of BHI cultures (37°C, aerobiosis) collected after 3, 4, 6, and 8 h of growth. **(A)** Kinetics of H_2_O_2_ production. **(B)** Amounts of H_2_O_2_ determined in 8 h cultures. **(C)** Kinetics of eDNA production. **(D)** Amounts of eDNA in 8 h cultures. Dots or columns represent means of data from three independent experiments; bars indicate standard deviations (*SD*). Asterisks indicate significant differences in relation to SK36 (Kruskal–Wallis with *pos hoc* Dunn’s test).

*Streptococcus sanguinis* SK36 produces H_2_O_2_ under aerobiosis, which in turn, promotes the production of eDNA by mechanisms not entirely understood (Kreth et al., 2009). Because eDNA is required for biofilm initiation in SK36 (Moraes et al., 2014), we compared kinetics of H_2_O_2_ and eDNA production in *S. sanguinis* strains grown in BHI. As shown in Figure 3A, all the strains produced H_2_O_2_, which increased in concentration until 6–8 h. Hydrogen peroxide achieved similar concentration at 8 h of growth in most strains, except in strain SK678, which showed reduction in the production of H_2_O_2_ (Figure 3B). Of note, SK678 showed reduced capacity to initiate biofilms (Figure 1). Kinetics of eDNA production during these same culture conditions were also assessed and showed increasing levels of eDNA mostly from 4 to 8 h of growth (increases from 9.91 to 2345.6 ng/ml) (Figure 3C). However, amounts of eDNA in 8 h cultures were more variable between strains, ranging from 28.66 to 2345.6 ng/ml (Figure 3D), and were compatible with profiles of H_2_O_2_ production, although could not be significantly correlated with amounts of H_2_O_2_ produced at this same time point (Pearson correlation; *r*: 0.417, *p* = 0.264). Strain SK160 produced atypical high amounts of eDNA (more than 2,000 ng/ml), although final concentration of H_2_O_2_ produced by this strain were similar to that produced by most strains tested (Figure 3B). Cultures of SK678 (a low H_2_O_2_ producer) yielded reduced mounts of eDNA. However, low eDNA yield was also observed in strains which produce significant amounts of H_2_O_2_, for example, SK115, which was also defective in biofilm initiation. Therefore, although the production of H_2_O_2_ is a conserved function of *S. sanguinis*, few strains differ in amounts of H_2_O_2_ produced in a fashion compatible with profiles of eDNA production and biofilm initiation phenotypes.

### *Streptococcus sanguinis* Strain Diversity in C3b Deposition Is Associated With the Susceptibility to Phagocytosis by Human PMN and Influenced by Growth Media and Sucrose-Derived Exopolysaccharides

**FIGURE 4:**
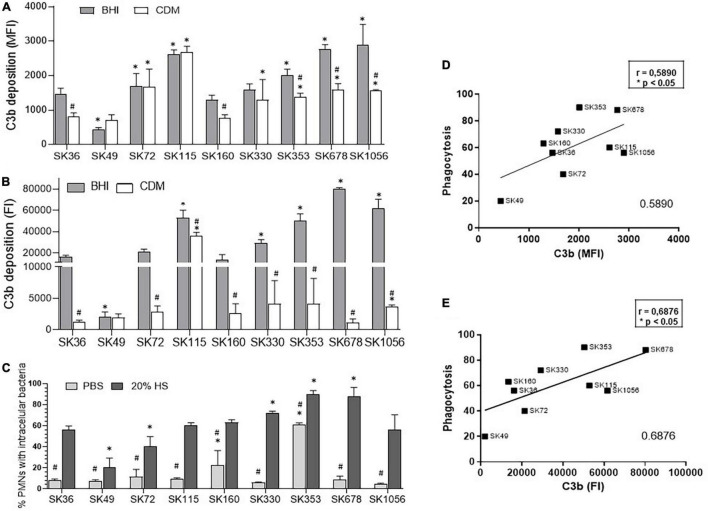
Profiles of C3b deposition and phagocytosis by PMN in *S. sanguinis* strains. These strains were grown in either BHI or CDM until mid-log phase of growth (A_550 nm_) were treated with 20% human serum (HS) for quantification of surface bound C3b by flow cytometry. **(A)** Relative levels of C3b deposition expressed as geometric means of fluorescence intensity (MFI). **(B)** Relative levels of C3b deposition expressed as fluorescence index (FI; the percentage of C3b-positive cells multiplied by the respective MFI values). Columns represent means of data obtained in three independent experiments; bars indicate *SD*. Asterisk indicates significant difference in relation to SK36 at the same tested condition; hashtag represents difference in C3b measures between strains grown in CDM medium compared to the same strain grown in BHI (Kruskal–Wallis with *post hoc* Dunn’s test). **(C)** The frequencies of bacterial phagocytosis were determined by the flow cytometry in PMN isolated from human peripheral blood and exposed to FITC-labeled strains in the absence or presence of 20% HS. Asterisk indicates significant difference in relation to SK36 at the same condition; hashtag indicates significant difference in the frequency of phagocytosis in the presence of HS compared to the same strain co-cultivated with PMN in the absence of HS (PBS) (Kruskal–Wallis with *post hoc* Dunn’s test). **(D,E)** Pearson correlation analyses between the levels of C3b-binding assessed in MFI and FI with the frequencies of serum-mediated phagocytosis by PMN.

*Streptococcus sanguinis* strains show reduced binding to C3b (a central effector component of the complement system) when compared to other commensal species of oral streptococci, but a diversity in C3b binding was detected in this species (Alves et al., 2019). In *S. mutans*, C3b deposition is impaired by surface-bound glucan EPS and it seems to be also influenced by components of BHI medium (Alves et al., 2016). Thus, we firstly investigated the effects of BHI in *S. sanguinis* binding to C3b, by comparing levels of surface bound C3b after serum treatment of strains previously grown in BHI and in CDM. Significant inter-strain variation was detected in C3b deposition as expressed in MFI or FI (Figures 4A,B). Although inter-strain variation in C3b binding observed in BHI-grown strains was comparable to variations in CDM-grown strains, intensities of C3b deposition were significantly higher in BHI- than in CDM-grown bacteria for several strains (Figures 4A,B), suggesting that BHI components adsorbed to bacterial cells may favor C3b deposition. Increases in C3b deposition in BHI-grown compared to CDM-grown strains were more evident when surface-bound C3b was measured using FI (Figure 4B), because this index reflects the intensities of C3b deposition, as well as the proportion of C3b-positive cells, being more accurate than MFI for inter-strain comparisons. Because, C3b is a major opsonin recognized by PMN, we further investigated if differences in C3b deposition in BHI-grown bacteria treated with serum were associated with the frequencies of phagocytosis of strains by PMN isolated from human peripheral blood in serum-dependent way. As shown in Figure 4C, phagocytosis of all *S. sanguinis* strains, except for strains SK353 and SK160, did not occur in the absence of human serum, indicating that *S. sanguinis* phagocytosis is mostly dependent on serum. Importantly, the inter-strain variations in C3b-binding (measured either in MFI or FI) were significantly associated with frequencies of phagocytosis by PMN (Figures 4D,E).

To further access the effects of sucrose-derived EPS in *S. sanguinis* evasion of C3b deposition and phagocytosis, intensities of C3b binding were compared in a sub-set of strains grown either in CMD or CDM supplemented with 1% sucrose (CDMS). As shown in Supplementary Figures 2A,B, the growth in CDMS significantly reduced the C3b deposition (measured either in MFI and/or FI) in strains SK36, SK330, SK678, and SK1056, when compared to these respective strains grown in CDM. On the other hand, C3b deposition was not affected by the medium supplementation with sucrose in SK72 and SK160. Importantly, changes in the C3b deposition were consistent with changes in serum-mediated phagocytosis accessed in the same culture conditions (Supplementary Figure 2C). As expected, phagocytosis was limited in the absence of serum when strains were either grown in CDM or CDMS (Supplementary Figure 2C). These findings indicate that sucrose-derived EPS not only promotes the biofilm maturation, but also impairs the C3b-mediated opsonophagocytosis by human PMN in most strains, including blood isolates (such as SK678 and SK1056).

### Susceptibilities to C3b Deposition Are Associated With Strain-Specific Profiles of Binding to C1q and SAP

**FIGURE 5:**
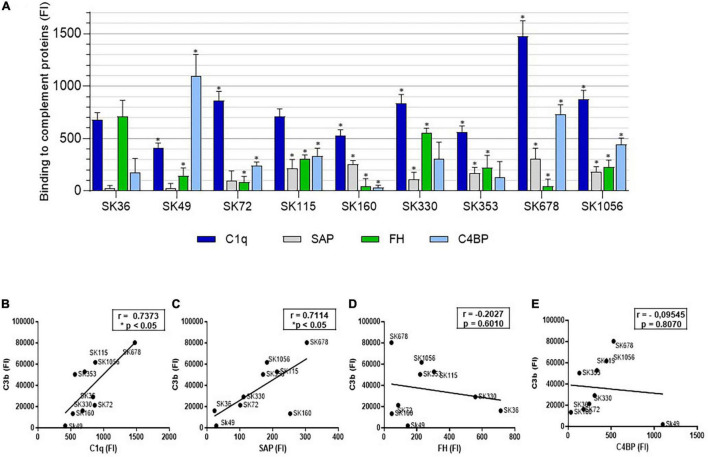
Comparative analysis of *S. sanguinis* strain binding to proteins for activation or regulation of the complement system. **(A)** Strains harvested from BHI cultures were treated with 20% human serum (37°C, 30 min.) and surface bound serum proteins probed with FITC-conjugated antibodies specific to human C1q, SAP, FH or C4BP for flow cytometry analysis. Intensities of binding to each protein were expressed using FI. Strains treated with PBS were used as negative controls. Columns represent means of data obtained in three independent experiments. Asterisks indicate significant differences in relation to SK36 for each respective human protein (Kruskal-Wallis with *post hoc* Dunn’s test; *p < 0.05). **(B–E)** Pearson correlation analyses between FI measures of binding to C1q, SAP, FH and C4BP respectively, with FI measures of C3b binding determined in the same culture conditions; asterisks indicate significant correlation (*p* < 0.05).

To further explore the potential mechanisms underlying inter-strain diversity in *S. sanguinis* binding to C3b, we investigated the binding of *S. sanguinis* strains to serum components involved in complement activation [C1q and the pentraxin serum amyloid P component (SAP)], as well as to the fluid-phase complement downregulators [Factor H (FH) and the C4b-binding protein (C4BP)]. As shown in Figure 5A, strains significantly differ in binding to these components. Importantly, strain binding to C3b was positively associated with the binding to the initial recognition proteins of the classical pathway of complement activation, C1q (Figure 5B) and SAP (Figure 5C). In addition, although no significant associations between C3b deposition and binding to C4BP or FH were detected in our correlation analyses (Figures 5D,E), strain SK49 which clearly evades C3b deposition and opsonophagocytosis by PMN, showed increased binding to C4BP compared to SK36 (Figure 5A). Therefore, variation in *S. sanguinis* strain binding to complement activators and/or fluid-phase regulators are associated with strain susceptibility to C3b deposition.

### *Streptococcus sanguinis* Strains Differ in Invasiveness Into Human Coronary Artery Endothelial Cells

**FIGURE 6:**
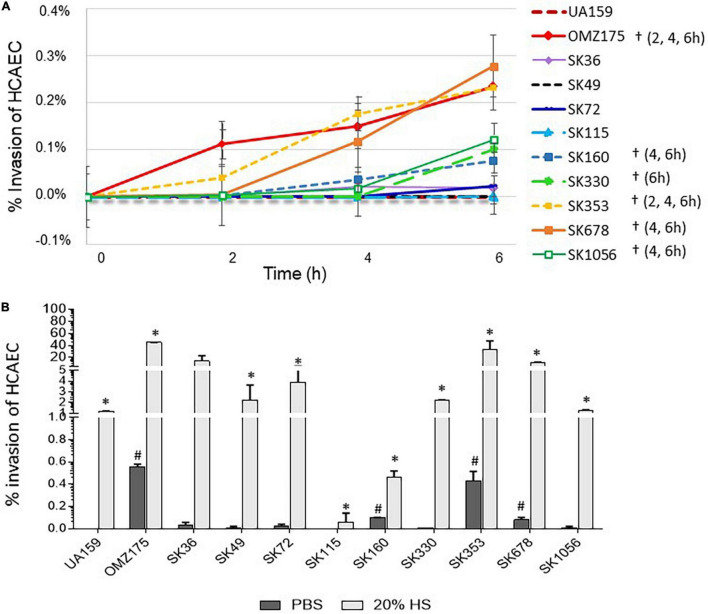
Comparisons of the *S. sanguinis* strain capacities to invade primary human coronary artery endothelial cells (HCAEC). **(A)** Kinetics of bacterial invasion into HCAEC. Invasion rates were expressed as the percentages of intracellular bacteria compared to initial inoculum, as determined in antibiotic-protection assays performed at MOI 100:1. Symbols (†) indicate significant differences in invasion rates in relation to SK36 at the respective time-points (indicated within parenthesis for each strain). **(B)** Analysis of the effects of binding to HS components in strain invasives. Antibiotic protection assays were performed with strains previously treated with 20% of HS or PBS (negative control), before co-cultivation with HCAEC during 2 h. Columns represent means of data obtained in three independent experiments. Symbols indicate significant difference in the percentages of invasion after treatment with HS (*) or with PBS (#) in relation to SK36 (Kruskal–Wallis with *post hoc* Dunn’s test; *p* < 0.05). The *Streptococcus mutans* strains UA159 and OMZ175 were used as reference in all the assays.

Bacterial invasion and persistence into endothelial cells are important mechanisms of pathogenicity in cardiovascular and other systemic infections (Kozarov, 2012; Konradt and Hunter, 2018). Thus, we investigated the capacities of *S. sanguinis* strains to invade primary HCAEC. Initially, we defined the kinetics of invasion of primary HCAEC by *S. sanguinis* strains in the absence of human serum, using the invasive and non-invasive *S. mutans* strains (OMZ175 and UA159, respectively) as reference (Abranches et al., 2011; Alves et al., 2020). As expected, OMZ175 showed increased invasion from 2 to 6 h of incubation with HCAEC, achieving the highest invasion percentages, whereas UA159 was not invasive (Figure 6A). Two *S. sanguinis* strains (SK678 and SK353) showed invasion kinetics similar to that of OMZ157. In addition, strains SK1056, SK330, and SK160 also showed frequencies of HCAEC invasion which were significantly higher than invasion rates of SK36 (Figure 6A). The strain SK115 was not able to invade HCAEC, similar to that observed for non-invasive UA159. We next investigated the influence of human serum in strain invasiveness, by exposing HCAEC cells to *S. sanguinis* strains previously treated with 20% human serum. As shown in Figure 6B, for most of the strains tested, the pre-treatment with human serum increased the invasiveness in approximately 100- to 600-fold at 2 h of bacterial challenge in relation to serum-independent rates of invasion. Serum treatment promoted, however, moderate effects on SK160 and SK115 invasiveness, implying defects of these strains in endothelial cell invasion or in intracellular persistence (Figure 6B). As verified in the absence serum, strains SK678 and SK353 showed the highest invasiveness in the presence of serum, reaching percentages of invasion similar to that found in OMZ175 (Figure 6B).

### Differences in *S. sanguinis* Strain Invasiveness Into Human Coronary Artery Endothelial Cells Are Associated With Diversity in Binding to Complement and Plasma Glycoproteins

**FIGURE 7:**
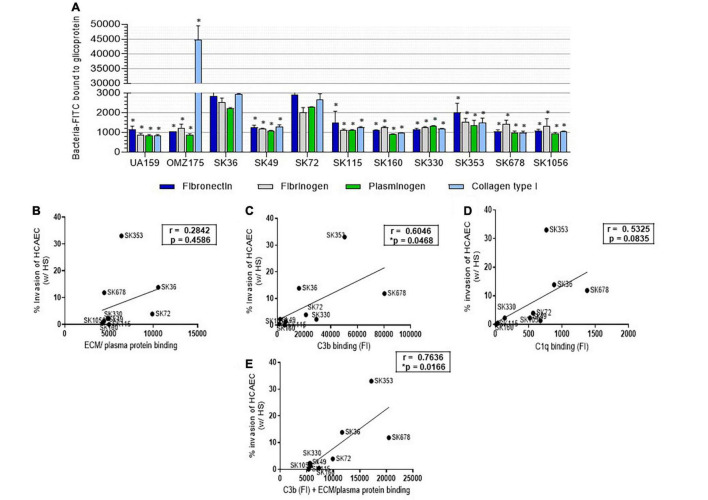
*Streptococcus sanguinis* strain binding to human proteins and correlations with invasion into HCAEC. **(A)** Strain binding to ECM/plasma human proteins was determined in microplate-based fluorescence assays. Microtiter plates coated with human fibronectin, fibrinogen, plasminogen, or collagen type I were incubated with FITC-labeled bacteria, and relative measures of binding determined using a fluorescence reader. Plates coated with BSA were used as controls. The *S. mutans* strains UA159 and OMZ175 were used as reference. Columns represent means of data of three independent experiments; asterisk indicates significant difference in relation to SK36 binding to the same protein (Kruskal–Wallis with *post hoc* Dunn’s test; *p* < 0.05). **(B–D)** Pearson correlation between invasion rates into HCAEC (after treatment with HS during 2 h of co-cultivation) with scores of binding measures to ECM/plasma human proteins (fibronectin, fibrinogen, plasminogen, and collagen type I), or with measures of binding to complement protein C3b and C1q, respectively. **(E)** Pearson correlation between HS-treated strain invasion into HCAEC with scores of binding to ECM/plasma plus C3b binding measures (FI). Asterisk indicates significant correlation at *p* < 0.05.

Endothelial cells dynamically interact with glycoproteins present in soluble form in plasma and as structural components of sub-endothelial ECM (Davis and Senger, 2005), which in turn, may function as receptors for bacterial binding and invasion. Thus, as a first step to investigate the potential mechanisms involved in *S. sanguinis* invasiveness into HCAEC, we screened for phenotypes of binding to plasma/ECM glycoproteins reported to modulate bacterial invasiveness, such as fibronectin, fibrinogen, plasminogen, and collagen type I (Lemichez et al., 2010; Abranches et al., 2011; Singh et al., 2012; Alves et al., 2020; Nomura et al., 2020). The *S. mutans* strains UA159 and OMZ175 were also used as reference for these phenotypes (Avilés-Reyes et al., 2014; Alves et al., 2020). As shown in Figure 7A, SK36 and SK72 showed the highest binding to fibronectin, fibrinogen, and plasminogen among all the *S. sanguinis* and *S. mutans* strains. Strains SK36 and SK72 also showed significant binding to type I collagen, but none of the *S. sanguinis* strains achieved intensities of collagen binding observed for OMZ175, which expresses the collagen-binding protein Cnm (Nomura et al., 2012; Avilés-Reyes et al., 2017). The other *S. sanguinis* strains showed similar profiles of binding to the tested glycoproteins. No significant associations were found between rates of serum-dependent invasion with each individual plasma/ECM glycoprotein tested (data not shown) or with scores of binding intensities to all the glycoproteins analyzed (sum of relative intensities of binding to the four glycoproteins) (Figure 7B). Because EC, such as HCAEC, expresses the receptors for C3b and C1q (Soares and Bach, 1999; Langeggen et al., 2002; Fischetti and Tedesco, 2006; Oroszlán et al., 2007; Yin et al., 2008), we further investigated the associations between invasiveness into HCAEC with intensities of binding to C3b and C1q. As shown in Figure 7C, serum-dependent invasiveness positively correlated with C3b binding. In addition, strain binding to C1q tended to correlate with strain invasiveness, although correlation did not achieve statistical significance (*p* = 0.08) (Figure 7D). Finally, scores of FI measures of C3b binding added of scores of bindings to the four glycoproteins (Figure 7E) showed increased correlation with bacterial invasiveness (*r* = 0.76; *p* < 0.05; Figure 7E), suggesting that strain-specific interactions with complement and ECM glycoproteins modulates the capacity to invade EC.

## Discussion

*Streptococcus sanguinis* strains isolated from children and adults show high genotypic and phenotypic diversity (Pan et al., 2001; Kreth et al., 2017; Baker et al., 2018), highlighting the need for addressing intra-species functional diversity to understand *S. sanguinis* behaviors as a beneficial commensal bacterium and/or systemic pathogen. Strain-specific genes associated with systemic virulence remains to be investigated in *S. sanguinis* strains (Baker et al., 2018). Moreover, virulence capacity of streptococci further relies on the strain-specific upregulation of conserved virulence genes, apparently due to polymorphisms in transcription regulators of virulence (Mattos-Graner and Duncan, 2017; Wilkening and Federle, 2017; Oliveira et al., 2021). In this study, we show that *S. sanguinis* strains largely differ in multiple phenotypes important for oral colonization and/or systemic virulence, such as biofilm formation, evasion to complement-mediated immunity, and invasion of primary HCAEC, clearly demonstrating that phenotypic observations on bacterial species need to be confirmed in multiple strains. We further show that binding to sucrose-derived EPS reduces *S. sanguinis* susceptibility to complement immunity, whereas strain-specific binding to SAP and C1q is associated with increased C3b deposition. Finally, we provide evidence that C3b deposition might function as a double-edged sword in immune surveillance of *S. sanguinis*, favoring phagocytosis by PMN, but also *S. sanguinis* invasion into endothelial cells, an issue that remains to be investigated in more detail.

Clinical and molecular studies indicate that *S. sanguinis* is competitive in initiating biofilms on saliva/plasma-coated teeth (Nyvad and Kilian, 1987; Kreth et al., 2005; Diaz et al., 2006; Bin et al., 2018), although the mechanisms through which *S. sanguinis* contributes to biofilm initiation and maturation were mostly assessed in SK36 (Bin et al., 2018). Divergence between biofilm initiation and maturation phenotypes observed in some strains strengthens the notion that multiple molecular mechanisms underlie the complex processes of biofilm initiation and maturation. Here, we identified defects in biofilm initiation in strains with low production of eDNA, consistently with the roles eDNA in biofilm initiation reported in SK36 (Moraes et al., 2014). On the other hand, biofilm maturation was also markedly affected by the presence of saliva in a strain-specific fashion. In *S. mutans*, saliva-mediated aggregation involving sortase-anchored surface proteins inhibits the biofilm maturation (Ahn et al., 2008), and an analogous process might explain the inhibitory effects of saliva on the biofilm maturation observed in specific *S. sanguinis* strains, because sortase-anchored surface proteins mediate *S. sanguinis* binding to salivary components (Kreth et al., 2017). On the other hand, in the absence of saliva, biofilm maturation was increased with the presence of sucrose, except for strains defective in EPS-mediated aggregation. Previous studies indicate that *S. sanguinis* GtfP supports biofilm maturation in strains capable of binding to to glucan (Yoshida et al., 2014; Liu et al., 2017; Bin et al., 2018), apart from the reduced stability of GtfP-derived glucan when compared to insoluble glucan produced by *S. mutans* GtfB/C (Kopec et al., 2001; Yoshida et al., 2014). In *S. mutans* and *Streptococcus mitis*, strains isolated from the systemic infections show increased binding to glucan produced by *S. mutans*, and thus, increased protection against C3b deposition (Alves et al., 2016; Harth-Chu et al., 2019). Here, we show that EPS produced by several *S. sanguinis* strains (such as the blood isolates SK678 and SK1056) clearly affected the serum-mediated phagocytosis by human PMN, further emphasizing the need to investigate mechanisms involved in *S. sanguinis* binding to EPS.

In the presence of oxygen, *S. sanguinis* expresses *spxB* encoding for a pyruvate oxidase, which synthesizes H_2_O_2_ (Redanz et al., 2018). Here, we show that most strains produce similar levels of H_2_O_2_ under aerobic conditions, compatible with the important roles of H_2_O_2_ production in *S. sanguinis* capacity to competitively initiate the biofilms in an eDNA-dependent fashion (Kreth et al., 2009; Moraes et al., 2014; Redanz et al., 2018). Deletion of *spxB* further reduces the SK36 *ex vivo* survival in human blood and increases its killing by PMN (Sumioka et al., 2017). However, we found that blood strain SK678 produced reduced levels of H_2_O_2_, and consistently, reduced amounts of eDNA and defects in biofilm initiation. This strain also showed increased growth in aerated medium in relation to SK36 (Supplementary Figure 1C) compared to static incubation (Supplementary Figure 1A), which might suggest increased capacity to cope with oxygen-dependent host defenses. High levels of H_2_O_2_ and eDNA were by contrast found in SK160, which showed robust capacity to initiate and to mature biofilms regardless the presence of saliva. Of note, SK160 was isolated from initial dental biofilms (Nyvad and Kilian, 1987; Kilian et al., 1989). Thus, variation in H_2_O_2_, although restricted to few strains, is compatible with diversity in the production of eDNA and with biofilm phenotypes. Further analysis of eDNA production in the presence of catalase would be required to investigate additional H_2_O_2_-independent mechanisms of DNA release in these strains.

Bacterial binding to C3b occurs through cleavage of serum C3 by C3-convertases (serine proteases) formed during three major activation cascades, the classical, the lectin and the alternative pathways (Dunkelberger and Song, 2010). Surface-bound C3b is a major opsonin for PMN phagocytosis of oral streptococci (Alves et al., 2016, 2019) and is required for the blood clearance by erythrocytes, as reported in *Streptococcus pneumoniae* (Li et al., 2007). Complement activation also generates active molecules for recruiting and/or activating innate and adaptive immune functions, which modulate chronic diseases, including atherogenesis (Lappegård et al., 2014; Ruan and Gao, 2019). Previously, we reported that *S. sanguinis* strains generally have low susceptibility to C3b deposition when compared to other species of oral streptococci (Alves et al., 2019). Here, we explored potential mechanisms involved in complement evasion by *S. sanguinis*. Firstly, we showed that for most strains (such as the blood strains SK678 and SK1056), C3b deposition was increased when bacteria were grown in BHI when compared to CDM, indicating that BHI components adsorbed to strains favor complement activation. However, we could not detect clear diversity in strain interactions with host glycoproteins present in plasma and/or ECM known to influence on complement activation (plasminogen and fibrinogen) and/or in interaction with host cells (e.g., fibronectin and collagen) (Hindmarsh and Marks, 1998; Carlsson et al., 2005; Barthel et al., 2012; Liu et al., 2018). On the other hand, strains differed in binding to C1q, a major pattern recognition protein of the complement classical pathway (Nayak et al., 2012), and importantly, C1q binding correlated with the levels of C3b deposition. These findings support the role of the classical pathway in complement immune surveillance of *S. sanguinis* through C1q recognition. Because the studied *S. sanguinis* strains do not significantly differ in binding to IgG from human serum pools (Alves et al., 2019), we hypothesized that differences in C1q binding could be mediated by the strain interactions with surface-bound antibody-like pentraxins present in serum, which include acute-phase C-reactive protein (CRP) and SAP (Nayak et al., 2012; Ma and Garred, 2018). *S. sanguinis* strains, however, do not bind CRP (Alves et al., 2019), but, here, we identify significant diversity in binding to SAP, which further correlated with C3b deposition. Also interesting was the high affinity of SK49, which is resistant to C3b deposition, to C4BP, a major fluid-phase downregulator of the classical and lectin pathways (Ermert and Blom, 2016). Thus, the strain-specific interactions with host pentraxins or fluid-phase complement downregulators seem to influence on *S. sanguinis* susceptibility to complement deposition. The molecular basis of C1q, SAP, and C4BP binding remains to be addressed.

Oral streptococci differ in their capacity to invade EC, and increased invasiveness is associated with systemic virulence (Stinson et al., 2003; Abranches et al., 2011; Nomura et al., 2020). We found marked differences in *S. sanguinis* strain invasiveness and identified a highly invasive strain isolated from the bloodstream, SK678. Although serum-independent invasion phenotypes were clearly less efficient than serum-mediated invasion, strain variation observed in the absence of serum proportionally reflected rates of serum-dependent invasion. Because strain invasiveness was associated with the C3b binding, we hypothesized that the invasion in the absence of human serum could be mediated by the production of C3/C1q by HCAEC itself. Endothelial cells, including HCAEC, express the complement proteins C3 and C1q, as well as receptors for C3b and C1q (Langeggen et al., 2000, 2002; Bossi et al., 2014). Moreover, EC binding to C1q-and/or C3b-linked antigens drives multiple endothelial responses involved in tissue protection, repair and angiogenesis (Soares and Bach, 1999; Oroszlán et al., 2007; Yin et al., 2008; Bossi et al., 2014). Interestingly, our observed profiles of strain invasiveness were consistent with rates of strain persistence in heart valve vegetations reported in a screening of 20 *S. sanguinis* strains using a rabbit model of infective endocarditis (Baker et al., 2018). Among those, SK678 was recovered at high abundance from infected heart valve vegetations followed by SK160, SK36, and SK353 (Baker et al., 2018), whereas poorly invasive strains in our assays (SK115, SK49, and SK330) were also less efficiently recovered from heart tissues (Baker et al., 2018). These findings reinforce the need for identifying *S. sanguinis* proteins required for the complement-mediated EC invasion and intracellular persistence, a topic of our future studies.

In summary, this study shows significant intra-species diversity in functions important for *S. sanguinis* capacities to initiate and maturate biofilms and to promote the cardiovascular infections, highlighting the need to explore the molecular mechanisms of host persistence and systemic virulence in a broader number of strains. Associations between strain binding to complement proteins with invasion into EC further open new venues to explore mechanisms of systemic virulence of this abundant species of the oral cavity.

## Data Availability Statement

The raw data supporting the conclusions of this article will be made available by the authors, without undue reservation.

## Ethics Statement

The studies involving human participants were reviewed and approved by the Ethics Committee of the Piracicaba Dental School, State University of Campinas (CEP/FOP-UNICAMP; protocol N°. 3.365.892; CAAE: 83140418.0.0000.5418). The patients/participants provided their written informed consent to participate in this study.

## Author Contributions

RM-G, LA, and TA conceived and designed the experiments. GS and LA performed the analyses of planktonic growth, biofilm, and aggregation phenotypes, and production of H_2_O_2_ and eDNA. LA, VF, and DB performed the analyses of bacterial binding to complement proteins. LA and TA performed the analysis of bacterial invasion into endothelial cells. LA analyzed the bacterial binding to host proteins. RM-G, LA, GS, VF, and JH analyzed and interpreted the data. RM-G and LA wrote the manuscript. All authors revised the manuscript and approved its final version.

## Conflict of Interest

The authors declare that the research was conducted in the absence of any commercial or financial relationships that could be construed as a potential conflict of interest.

## Publisher’s Note

All claims expressed in this article are solely those of the authors and do not necessarily represent those of their affiliated organizations, or those of the publisher, the editors and the reviewers. Any product that may be evaluated in this article, or claim that may be made by its manufacturer, is not guaranteed or endorsed by the publisher.

## Supplementary Material

Supplementary Figure 1 Growth curves of *Streptococcus sanguinis* strains under different atmospheric conditions. These strains were grown in BHI (37°C) at two atmospheric conditions. **(A,B)** Static incubation (10% CO_2_, 90% air). **(C,D)** Aerobic shaking (160 rpm). Absorbance measures represent means of three experiments; bars indicate standard deviation. Growth curves of strains were compared with the reference strain SK36 (black continuous line). Asterisks indicate statistically significant differences in absorbance values (A_550 nm_) in relation to SK36 strain at the same incubation time (Mann–Whitney *U*-test; *p* < 0.05).

Supplementary Figure 2 Effects of sucrose-derived EPS in C3b deposition and phagocytosis by PMN in *S. sanguinis* strains. These strains grown either in CDM or CDM supplemented with 1% sucrose (CDMS) were treated with 20% human serum (HS) for determination of levels of C3b deposition by flow cytometry. These strains treated with PBS instead of HS were used as negative controls. **(A)** Relative levels of surface bound C3b were expressed as geometric mean fluorescence intensity (MFI). **(B)** Levels of surface bound C3b expressed as fluorescence index (FI; the percentage of C3b-positive cells multiplied by the respective MFI values). Columns represent means of data obtained in three independent experiments; bars indicate standard deviations. The asterisks symbol indicates significant difference in C3b measures in bacteria grown in CDMS compared to the same strain grown in CDM. **(C)** The frequencies of bacterial phagocytosis were determined by the flow cytometry of PMN isolated from human peripheral blood and exposed to FITC-labeled strains (from cultures obtained either in CDM or CDMS) in the absence or presence of 20% HS. The asterisks symbol indicates significant difference between the frequency of HS-mediated phagocytosis in strain from CDMS culture compared to the same strain grown in CDM. Hashtag symbol indicates significant difference between the frequency of HS-independent phagocytosis observed in strain from CDMS culture compared to the same strain grown in CDM.
