# Supplementary figures and images for: Diversity in Phenotypes Associated With Host Persistence and Systemic Virulence in Streptococcus sanguinis Strains
Source: Front Microbiol. 2022 Apr 18;13:875581. doi: 10.3389/fmicb.2022.875581 (PMC9058168; doi:10.3389/fmicb.2022.875581)

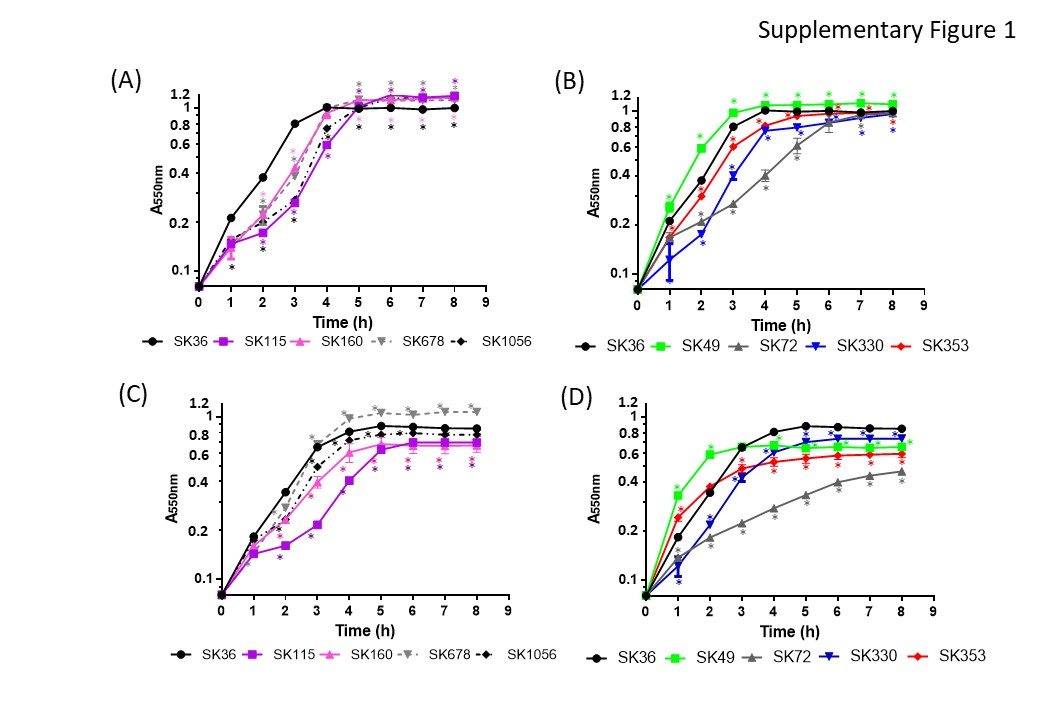

Supplement: Supplementary Figure 1 — Growth curves of Streptococcus sanguinis strains under different atmospheric conditions. These strains were grown in BHI (37°C) at two atmospheric conditions. (A,B) Static incubation (10% CO2, 90% air). (C,D) Aerobic shaking (160 rpm). Absorbance measures represent means of three experiments; bars indicate standard deviation. Growth curves of strains were compared with the reference strain SK36 (black continuous line). Asterisks indicate statistically significant differences in absorbance values (A550 nm) in relation to SK36 strain at the same incubation time (Mann–Whitney U-test; p < 0.05). [file Image_1.JPEG]

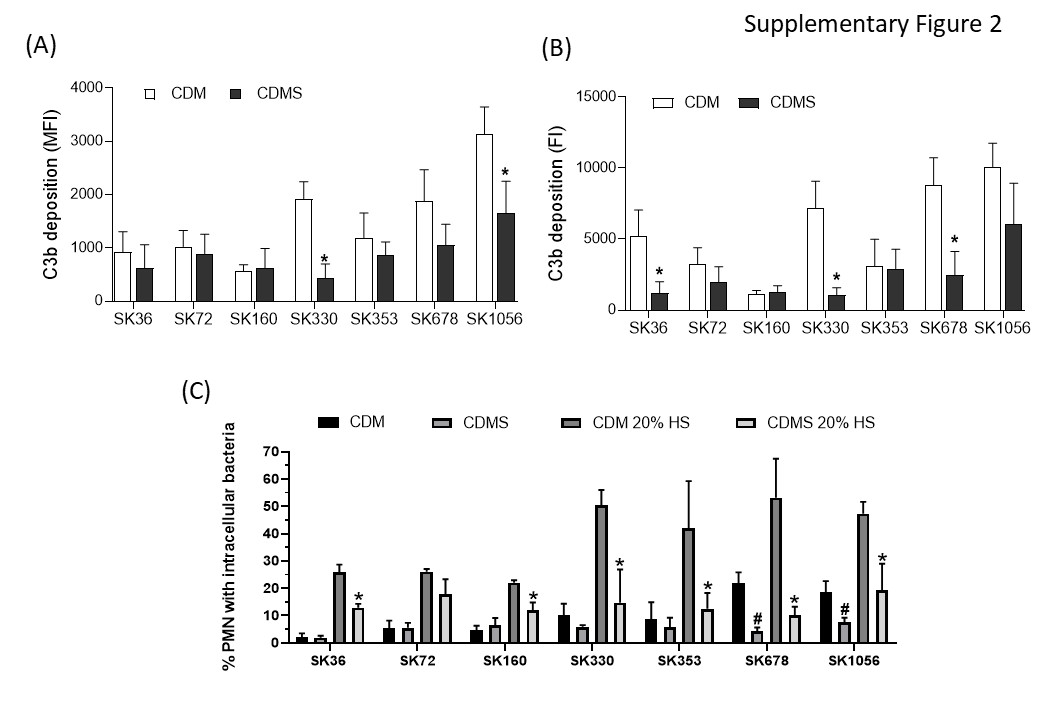

Supplement: Supplementary Figure 2 — Effects of sucrose-derived EPS in C3b deposition and phagocytosis by PMN in S. sanguinis strains. These strains grown either in CDM or CDM supplemented with 1% sucrose (CDMS) were treated with 20% human serum (HS) for determination of levels of C3b deposition by flow cytometry. These strains treated with PBS instead of HS were used as negative controls. (A) Relative levels of surface bound C3b were expressed as geometric mean fluorescence intensity (MFI). (B) Levels of surface bound C3b expressed as fluorescence index (FI; the percentage of C3b-positive cells multiplied by the respective MFI values). Columns represent means of data obtained in three independent experiments; bars indicate standard deviations. The asterisks symbol indicates significant difference in C3b measures in bacteria grown in CDMS compared to the same strain grown in CDM. (C) The frequencies of bacterial phagocytosis were determined by the flow cytometry of PMN isolated from human peripheral blood and exposed to FITC-labeled strains (from cultures obtained either in CDM or CDMS) in the absence or presence of 20% HS. The asterisks symbol indicates significant difference between the frequency of HS-mediated phagocytosis in strain from CDMS culture compared to the same strain grown in CDM. Hashtag symbol indicates significant difference between the frequency of HS-independent phagocytosis observed in strain from CDMS culture compared to the same strain grown in CDM. [file Image_2.JPEG]
